# Impact of intratumoral microbiome on tumor immunity and prognosis in human pancreatic ductal adenocarcinoma

**DOI:** 10.1007/s00535-023-02069-5

**Published:** 2024-01-20

**Authors:** Shohei Abe, Atsuhiro Masuda, Tomonori Matsumoto, Jun Inoue, Hirochika Toyama, Arata Sakai, Takashi Kobayashi, Takeshi Tanaka, Masahiro Tsujimae, Kohei Yamakawa, Masanori Gonda, Shigeto Masuda, Hisahiro Uemura, Shinya Kohashi, Noriko Inomata, Kae Nagao, Yoshiyuki Harada, Mika Miki, Yosuke Irie, Noriko Juri, Testuhisa Ko, Yusuke Yokotani, Yuki Oka, Shogo Ota, Maki Kanzawa, Tomoo Itoh, Toshio Imai, Takumi Fukumoto, Eiji Hara, Yuzo Kodama

**Affiliations:** 1 Division of Gastroenterology, Department of Internal Medicine, Kobe University Graduate School of Medicine, Kobe, Hyogo 650-0017 Japan; 2 Research Institute for Microbial Diseases, Osaka University, Suita, Osaka 565-0871 Japan; 3 Division of Hepato-Biliary-Pancreatic Surgery, Department of Surgery, Kobe University Graduate School of Medicine, Kobe, Hyogo 650-0017 Japan; 4 Division of Diagnostic Pathology, Kobe University Graduate School of Medicine, Kobe, Hyogo 650-0017 Japan

**Keywords:** Tumor microbiome, Prognosis, Tumor-infiltrating lymphocytes, Major driver gene alteration, Pancreatic ductal adenocarcinoma

## Abstract

**Background:**

Recent evidence suggests that the presence of microbiome within human pancreatic ductal adenocarcinoma (PDAC) tissue potentially influences cancer progression and prognosis. However, the significance of tumor-resident microbiome remains unclear. We aimed to elucidate the impact of intratumoral bacteria on the pathophysiology and prognosis of human PDAC.

**Methods:**

The presence of intratumoral bacteria was assessed in 162 surgically resected PDACs using quantitative polymerase chain reaction (qPCR) and in situ hybridization (ISH) targeting 16S rRNA. The intratumoral microbiome was explored by 16S metagenome sequencing using DNA extracted from formalin-fixed paraffin-embedded tissues. The profile of intratumoral bacteria was compared with clinical information, pathological findings including tumor-infiltrating T cells, tumor-associated macrophage, fibrosis, and alterations in four main driver genes (*KRAS*, *TP53*, *CDKN2A*/p16, *SMAD4*) in tumor genomes.

**Results:**

The presence of intratumoral bacteria was confirmed in 52 tumors (32%) using both qPCR and ISH. The 16S metagenome sequencing revealed characteristic bacterial profiles within these tumors, including phyla such as *Proteobacteria* and *Firmicutes*. Comparison of bacterial profiles between cases with good and poor prognosis revealed a significant positive correlation between a shorter survival time and the presence of anaerobic bacteria such as *Bacteroides*, *Lactobacillus*, and *Peptoniphilus*. The abundance of these bacteria was correlated with a decrease in the number of tumor-infiltrating T cells positive for CD4, CD8, and CD45RO.

**Conclusions:**

Intratumoral infection of anaerobic bacteria such as *Bacteroides*, *Lactobacillus,* and *Peptoniphilus* is correlated with the suppressed anti-PDAC immunity and poor prognosis.

**Supplementary Information:**

The online version contains supplementary material available at 10.1007/s00535-023-02069-5.

## Introduction

Pancreatic ductal adenocarcinoma (PDAC) is one of the most aggressive and lethal human cancers, with a 5-year survival rate of less than 10% [1]. PDAC progression and metastasis are not solely determined by genetic alterations in cancer genomes but also by the complex interplay between cancer cells and tumor microenvironment (TME), including tumor-infiltrating lymphocytes (TILs) and dense stromal fibrosis [2, 3]. TILs, particularly tumor-infiltrating CD8^+^ T cells, critically impact on the prognosis of PDAC as immune effectors capable of eliminating cancer cells [4]. Tumor stroma, densely populated with cancer-associated fibroblasts (CAFs) such as ACTA2 (α-SMA)^+^ myofibroblasts and collagen fibers, has also been reported to promote resistance to chemotherapy and pancreatic tumor progression by inducing hypoxic TME [5–7]. However, the factors that influence the formation of such pancreatic TME have been poorly understood.

Although the pancreas was previously supposed to be sterile, several recent studies have reported the presence of bacteria in the pancreatic tissues. Orally administered bacteria were reportedly detected in the mouse pancreas within 30 min [8], and correlation of the microbiome in the human duodenum and pancreas suggests that bacteria may directly translocate from the duodenum into the pancreas [9]. Furthermore, recent studies have confirmed the presence of bacteria within PDAC and its potential influence on tumor growth [10]. A mouse study demonstrated that microbiota in PDAC promotes oncogenesis by inducing innate and adaptive immune suppression. Corresponding relationship between microbiome and progressive PDAC suggested the oncogenic impact of intratumoral bacteria on PDAC development both in mice and humans [8]. However, another study reported that PDAC with more bacterial diversity showed enhanced immune infiltration and favorable prognosis compared with PDAC with less diverse bacteria [11]. Although tumor-resident bacteria in PDAC have been suggested to affect anti-tumor immunity and tumor prognosis, details remain controversial and unclear. In this study, we investigated bacteria within PDAC tissues that were surgically resected and aimed to elucidate the impact of intratumoral bacteria on TME and post-operative prognosis.

## Materials and methods

### Sample and data collection

We analyzed formalin-fixed paraffin-embedded (FFPE) sections from 162 PDAC patients who underwent surgery between April 2008 and March 2017 at the Kobe University Hospital. We retrospectively reviewed the patients’ medical information regarding age, gender, body mass index (BMI), carcinoembryonic antigen (CEA, reference range: < 5 ng/mL), and carbohydrate antigen 19-9 (CA19-9, reference range: < 37 ng/mL), white blood cell count, neutrophil count, total leukocytes count, alcohol consumption, current smoking, diabetes mellitus and administration of antibiotics. The tumor characteristics included tumor size and location, pathological stage (Union for International Cancer Control UICC] 8th classification), histological grade, residual tumor status after surgery, and the history of neoadjuvant and adjuvant chemotherapy. Laboratory data were collected within 1 month before surgery.

The study protocol was reviewed and approved by the ethics committee of the Kobe University Hospital (No.180235). Informed consent was waived due to the retrospective study design and the study information was disclosed on our hospital website, allowing eligible patients to opt out. This study was conducted in accordance with the Declaration of Helsinki. All authors had access to the study data, and reviewed and approved the final manuscript.

### DNA extracting and real-time PCR

DNA was extracted from the pancreatic tumoral and adjacent non-tumoral FFPE tissues using the QIAamp DNA FFPE Tissue Kit (Qiagen, Hilden, Germany) following the manufacturer’s protocol. The extracted DNA was quantified using a fluorometer (Invitrogen Qubit 4.0; Thermo Fisher Scientific, Waltham, MA, USA) and stored at −80 ℃ until further analysis.

Quantitative polymerase chain reaction (qPCR) was performed on Applied Biosystems 7500 real-time PCR system (Applied Biosystems Inc, CA, USA) using SYBR green qPCR assay (Applied Biosystems Inc, CA, USA). The V1–2 regions of 16S rRNA gene were amplified using forward primer 27Fmod (5′-AGRGTTTGATYMTGGCTCAG-3′), and reverse primer 338R (5′-TGCTGCCTCCCGTAGGAGT-3′) [12]. The cycling conditions were 1 cycle at 95 ℃ for 10 min to denature DNA, with amplification proceeding for 40 cycles at 95 ℃ for 15 s, 50 ℃ for 20 s, and 72 ℃ for 1 min, followed by a standard denaturation curve protocol.

### In situ hybridization (ISH)

Chromogenic RNA in situ hybridization (ISH) targeting 16S rRNA was performed using RNAscope^®^ 2.5 HD Reagent Kit-RED (Advanced Cell Diagnostics, Hayward, CA, USA) and Fast Red according to the manufacturer’s protocol. The probe used was RNAscope^®^ Probe-EB-16S-rRNA (Cat #464461). The chromogenic reaction within the pancreatic tumor indicated a positive result.

### Amplicon sequencing and microbiome analysis

The 16S rDNA V1–2 regions were amplified by PCR and sequenced in the MiSeq platform (Illumina) with MiSeq Reagent kit v2 (500 cycles) using the 250 bp paired-end protocol. The QIIME2 (version 2020.11) pipeline was used to perform microbiome analysis [13]. Demultiplexing and quality filtering were performed on the raw sequence data using the q2-demux plug-in, and amplicon sequence variants (ASVs) were counted after denoising by DADA2 [14].

Taxonomy was assigned to ASVs using reference sequences from Silva (138 SSURef NR99 full-length taxonomy). Bacterial contamination was distinguished using R program package “Decontam” with the parameter “method = frequency” by comparing the data from pancreatic tissues with that derived from 15 samples of FFPE pieces without tissues [15]. Alpha diversity analysis with Shannon index was calculated using QIIME2. Beta diversity analysis using weighted-UniFrac Principal Coordinate Analysis (PCoA) and permutation analysis of variance (PERMANOVA) were also performed using Qiime2. The taxonomic types of intratumoral microbiome to distinguish tumor prognosis were analyzed by linear discriminant analysis (LDA) effect size (LEfSe) calculations using Galaxy Version 1.0 [16].

### Immunohistochemistry (IHC) and Elastica van Gieson (EVG) staining

FFPE tissues were sectioned at 5 mm thickness and analyzed by IHC and EVG staining. The following antibodies were used: anti-TP53 (Santa Cruz Biotechnology, Dallas, TX, USA, catalog number: sc-47698), anti-CDKN2A/p16 (Roche Diagnostics, Cat #6695221001), anti-SMAD4 (Santa Cruz Biotechnology, Cat #sc-7966), anti-CD4 (Leica Biosystems, Wetzlar, Germany, Cat #CD4-368-L-CE), anti-CD8 (Roche Diagnostics, Cat #5493846001), anti-FOXP3 (Abcam, Cambridge, UK, Cat #ab20034), anti-CD45RO (BioGenex Laboratories, San Ramon, CA, USA,Cat #AM113-5M), anti-CD68 (ProteinTech Illinois, USA, Cat #66231-2-Ig), anti-CD206 (ProteinTech Illinois, USA, Cat #60143-1-Ig) and anti-α-SMA (Santa Cruz Biotechnology, catalog number: sc-53142). EVG staining was performed using an Elastic Stain Kit (Abcam, Cat #ab150667) according to the manufacturer’s protocol.

### Evaluation of tumor-infiltrating lymphocytes (TILs), macrophage, and tumor fibrosis

TILs positive for CD4, CD8, FOXP3, CD45RO and macrophage for CD68, CD206 were assessed by immunohistochemical staining on slides with the maximum divided surface of tumors. Each subset of TILs and macrophage were counted at 200 × magnification (counts/mm^2^) using Image J (Java image processing program inspired by National Institute of Health (NIH), USA). Three fields separated by at least 5 mm each were counted and the mean value was calculated for each case. The cases were classified as high density or low density based on the median value.

To assess fibrosis within pancreatic cancer, tumor stromal collagen and myofibroblasts were evaluated by EVG staining and immunostaining for α-SMA, respectively. The stained sections were digitally scanned and analyzed using Adobe Photoshop CC2019 software (Adobe Inc., San Jose, CA). The red area in EVG-stained sections and the brown area in α-SMA-stained sections were quantified as tumor stromal collagen and αSMA^+^ myofibroblasts, respectively. Each area was divided by the whole tumor area analyzed and defined as the area proportion of “tumor stromal collagen” and “α-SMA^+^ fibroblast,” respectively. Additionally, all cases were classified into two groups (high and low) based on the median value of the area proportion. Representative IHC images for TILs, CD68, CD206, α-SMA, and EVG staining image are shown in Supplementary Fig. 1.

### Evaluation of driver gene alterations

Alterations of *KRAS*, *TP53*, *CDKN2A/p16*, and *SMAD4* genes in the tumor were determined by next-generation sequencing (NGS) analysis, droplet digital PCR (ddPCR) and IHC using DNA extracted from FFPE as reported previously [17]. In brief, *KRAS* mutations were determined by NGS. *TP53* mutations were determined based on a combination of NGS, ddPCR, and IHC. *CDKN2A/p16* and *SMAD4* mutations were determined using IHC. IHC sections were evaluated by two experienced pathologists (M.K. and T.I.) who were unaware of the clinical data. *TP53*, *CDKN2A/p16*, and *SMAD4* were evaluated with Kappa values of 0.982 (*P* < 0.0001), 0.964 (*P* < 0.0001), 0.942 (*P* < 0.0001), respectively, and the agreement was high between the pathologists.

### Statistical analysis

SPSS (SPSS Inc., Chicago, IL, USA) and GraphPad Prism 8 (GraphPad Software, La Jolla, CA, USA) were used for statistical analyses. The chi-square test or Fisher’s exact test, when applicable, was used to compare frequencies, and the Wilcoxon rank-sum test was used to compare skewed continuous variables. Overall survival (OS) was estimated using the Kaplan–Meier method and compared using a log-rank test. Hazard ratios (HRs) and the corresponding 95% confidence intervals (CIs) were estimated using Cox proportional-hazards models. The multivariate analyses included factors with statistical significance in univariate analysis. All statistical tests were two-tailed, and statistical significance was set at *P* < 0.05.

## Result

### Identification of bacteria within PDAC

**Fig. 1:**
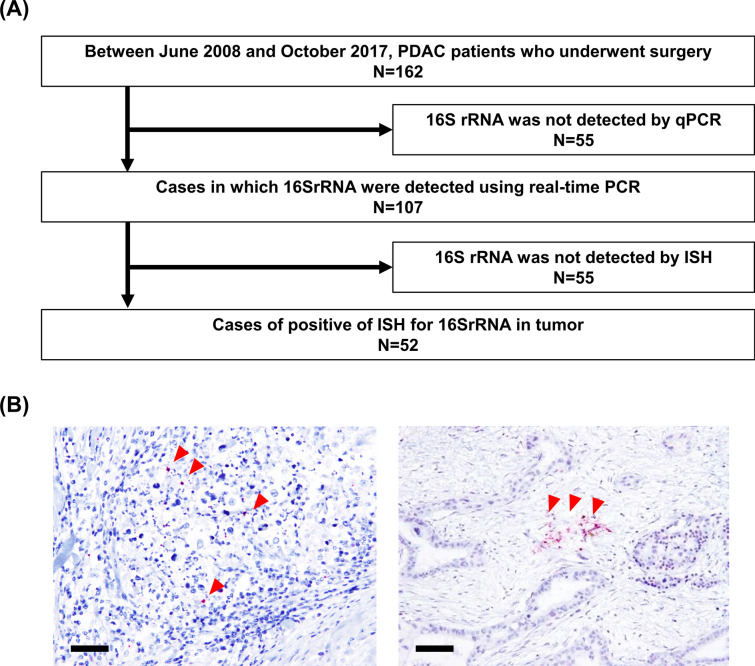
Study outline. **A** Flow chart to determine the presence of bacteria in human PDAC tissues. Of the 162 cases, 55 cases in which 16SrRNA could not be extracted by qPCR were excluded. In addition, of the remaining 107 cases, 55 cases in which 16SrRNA could not be identified in the tumor by ISH were excluded. Finally, 52 cases were included in this study. **B** Representative images of in situ hybridization targeting bacterial 16S rRNA gene. Red arrow heads indicate positive signals. Scale bars, 50 μm. *PDAC* pancreatic ductal adenocarcinoma, *PCR* polymerase chain reaction, *ISH* in situ hybridization

To identify tumors that contained intratumoral bacteria (Fig. 1A), we initially screened 162 human PDAC samples by qPCR targeting bacterial 16S rRNA gene, which detected positive amplification in 107 samples. These samples were further evaluated by ISH for 16S rRNA (Fig. 1B), and we confirmed 52 PDACs (32.1% among 162 tumors) that contained tumor-resident bacteria both on qPCR and ISH. Clinical information of the 52 cases with PDACs definitely positive for intratumoral bacteria were shown in Table 1.

**Table 1:** Patients’ characteristics strongly suggested presence of intra-tumor bacteria in PDAC

	Cases strongly suggested the presence of intra-tumor bacteria^a^
	*N* = 52
Age (years), median (range)	70 (50–83)
Gender, *n*	
Male/female	29/23
BMI (kg/m^2^), median (range)	20.9 (17.1–33.2)
CEA (ng/mL), median (range)	3.1 (0.1–68.8)
CA19-9 (U/mL), median (range)	163.5 (1.0–11973)
White blood cell count (/µL), median (range)	5600 (3600–8600)
Neutrophils count (/µL), median (range)	3199 (2113–6879)
Total leukocytes count (/µL), median (range)	1527 (749–2580)
Presence of alcohol consumption, *n* (%)	4 (7.7)
Current smoking, *n* (%)	10 (19.2)
Presence of diabetes melitus, *n* (%)	25 (48.1)
Administration of antibiotics, *n* (%)	11 (21.1)
Tumor size (mm), median (range)	29 (12–45)
Tumor location, *n*	
Head/body/tail	35/16/1
Pathological stage^b^, *n*	
IA/IB/IIA/IIB/III	7/5/5/28/7
Histological grade, *n*	
Well/moderate/poorly	16/33/3
Residual tumor status, *n*	
R0/R1/	38/14
Neoadjuvant chemotherapy, *n* (%)	5 (9.6)
Adjuvant chemotherapy, *n* (%)	37 (71.2)

*BMI* body mass index, *CEA* carcinoembryonic antigen, *CA19-9* carbohydrate antigen 19-9, *n* number of patients, *PDAC* pancreatic ductal carcinoma

^a^Cases exhibit positive results in both qPCR and ISH for 16SrRNA

^b^Pathological stage was classified according to the UICC 8th edition

**Fig. 2:**
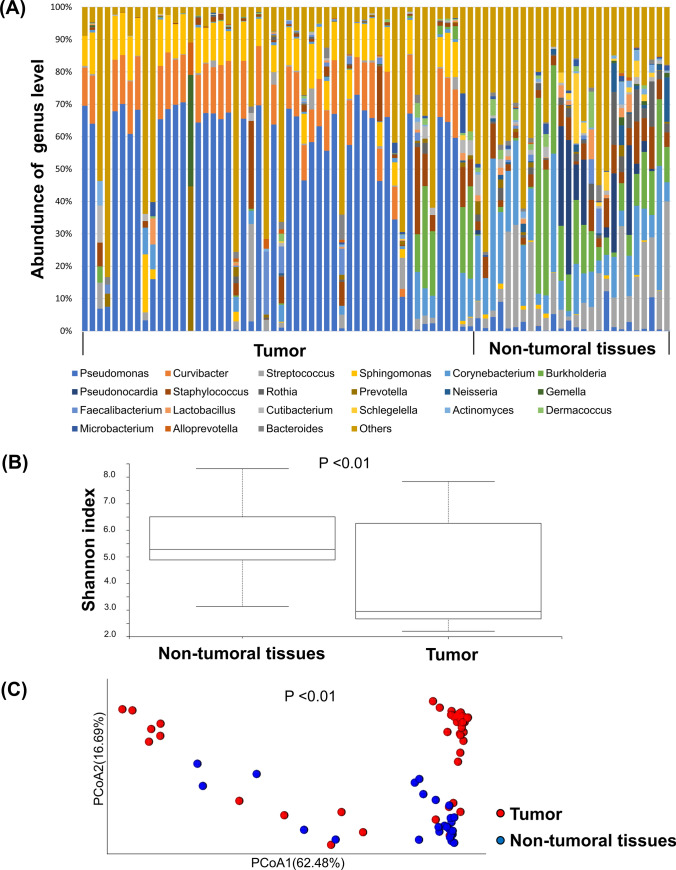
Microbiome composition in PDAC and non-tumoral tissues. **A** Taxonomic profiles of predominant bacterial genera by mean relative abundance (%) in PDAC tissue and non-tumoral tissues. **B** Comparative analysis of the alpha diversity of the microbiome communities between PDAC and non-tumoral tissues using Shannon index. **C** Principal coordinate analysis (PCoA) using weighted-UniFrac distance of beta diversity among PDAC and non-tumoral tissues. *PDAC* pancreatic ductal adenocarcinoma

To characterize the microbiome within PDAC, the 52 bacteria-positive PDACs were analyzed by 16S metagenome sequencing. As their non-tumoral counterparts, 26 non-tumoral tissues that were sufficiently distant from the tumor and positive for 16S rRNA amplification in qPCR were collected among the 52 cases with bacteria-positive PDAC and examined in parallel. To carefully exclude reads resulting from inevitable contamination of environmental bacteria, we assessed the sequencing data derived from FFPE tissues in comparison with those from non-tissue control pieces (see “Materials and methods”) using the R-package “Decontam.” The composition at the genus level before and after the use of Decontam are shown in Supplementary Fig. 2A and Fig. 2A, respectively. After the process excluding contamination with Decontam, many taxonomic levels of bacteria (1315 species, 723 genera, 306 families, 170 orders, 74 steels, 38 phylum) were identified in the tumor and non-tumoral tissues. At the phylum level (Supplementary Fig. 2B), *Proteobacteria* and *Firmicutes* were highly abundant in PDAC tissues, which was consistent with previous reports [10]. At the genus level, the genera *Pseudomonas*, *Curvibacter, Streptococcus*, *Sphingomonas*, and *Corynebacterium* were abundantly detected in PDACs (Fig. 2A). Subsequently, to investigate changes in the microbiome composition within PDAC due to administration of antibiotics, a diversity analysis was performed between 11 cases where antibiotics were used within 1 month before surgery and 41 cases where they were not used. There were no significant differences in alpha diversity measures by Shannon Index (*P* = 0.42) and beta diversity analyzed by weighted-UniFrac PcoA (*P* = 0.25, using PERMANOVA) in the two groups (Supplementary Fig. 3). These results indicate that the administration of antibiotics did not significantly alter the composition of the microbiome within PDAC in this study.

Alpha diversity measures by Shannon Index were significantly lower (*P* < 0.01) in PDAC than non-tumoral tissues (Fig. 2B). Beta diversity analyzed by weighted-UniFrac PcoA was also significantly different between PDAC and non-tumoral tissues (*P* < 0.01, using PERMANOVA) (Fig. 2C). These findings suggested that microbiome within PDAC tissues were unique and different from those in non-tumoral tissues. At least approximately one third of human PDACs were suggested to have tumor-resident bacteria, and our metagenome sequencing successfully detected the characteristics of microbiome within PDAC while eliminating the effects of contamination.

### Identification of PDAC microbiome associated with prognosis

**Fig. 3:**
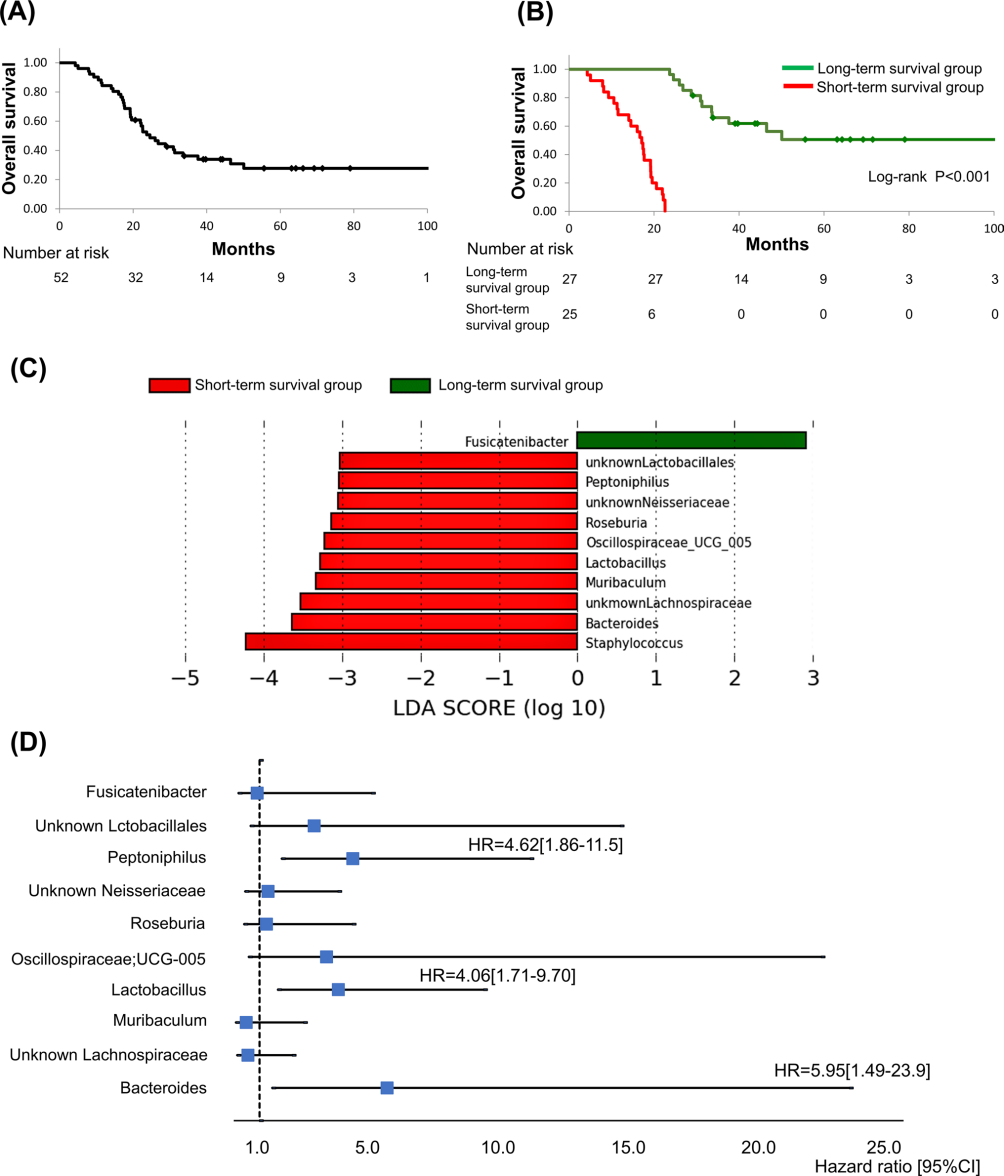
Identification of intratumoral bacteria associated with prognosis in pancreatic cancer patients. **A** Kaplan–Meier curve of overall survival of patients included in this study. **B** Overall survival of patients with PDAC in short-term survival group and long-term survival group. The patients were divided into two groups based on the median follow-up period (23.6 months). The survival curves were compared between them by the log-rank test. **C** LEfSe calculations between the short-term survival group and the long-term survival group were performed using a threshold of 2.0. **D** Forest plots showing the hazard ratio of prognosis by the presence of each genus detected by LEfSe. *PDAC* pancreatic ductal adenocarcinoma, *LEfSe* linear discriminant analysis effect size

To identify the intratumoral microbiome involved in prognosis, we first assessed the survival of the 52 patients with bacteria-infected PDAC (Fig. 3A). The median follow-up period of the cohort was 23.6 months, and a majority of the patients (71.2%, 37/52) did not survive during the follow-up period. The 52 patients were effectively divided into two groups based on the median follow-up period: “short-term survival group” and “long-term survival group” (Fig. 3B). All patients in the short-term survival group died within the observation period, and the overall survival between the two groups showcased significant difference (*P* < 0.001). A comparison of the patients’ backgrounds of the two groups showed significant differences in pathological stage and residual tumor status (Supplementary Table 1).

Subsequently, we compared the tumor-resident microbiome between the two groups. Alpha diversity tended to be lower in the long-term survival group, but was not significantly different (*P* = 0.06, using Shannon index). There was no significant difference in beta diversity between the two groups (*P* = 0.58, using permutational multivariate analysis of variance, Supplementary Fig. 4). LEfSe analysis was performed to explore the differences in the predominance of bacterial communities between the two groups, which demonstrated that one genus and ten genera were dominant in the long-term survival group and the short-term survival group, respectively (Fig. 3C). The presence of any of these genera identified other than Staphylococcus was associated with an increased HR for the prognosis of PDAC in the univariate analysis (Supplementary Table 2). Further multivariate analysis of the ten genera that were significantly different in the univariate analysis revealed that the presence of *Bacteroides*, *Lactobacillus*, and *Peptoniphilus* were significantly associated with poor prognosis (Fig. 3D).

### Association of intratumoral bacteria with prognostic factors in PDAC

**Fig. 4:**
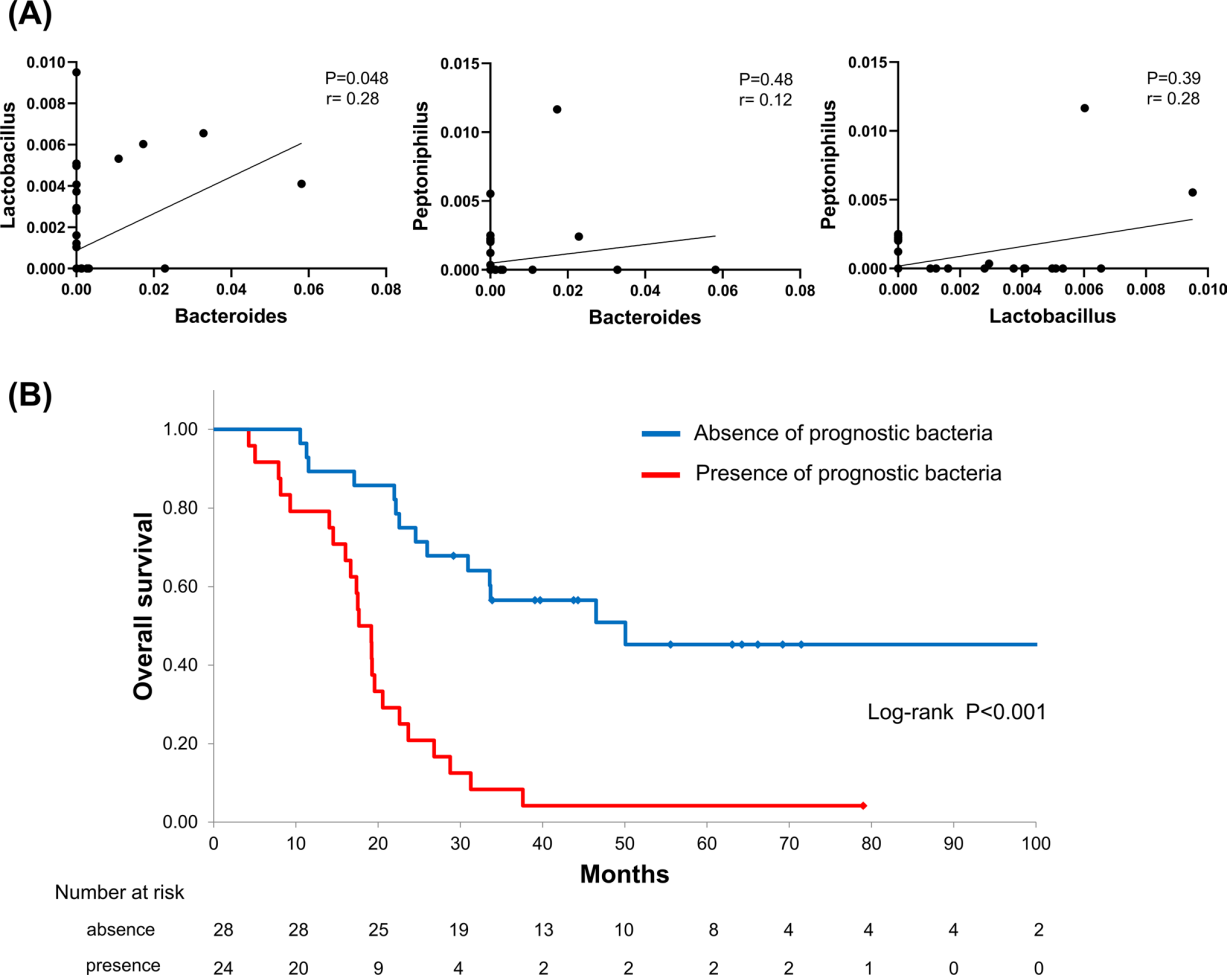
Correlations among prognostic bacteria and tumor prognosis. **A** Spearman correlation between *Bacteroides*, *Lactobacillus*, and *Peptoniphilus*. **B** Kaplan–Meier curve of overall survival of patients with and without prognostic bacteria. *PDAC* pancreatic ductal adenocarcinoma

Notably, the three genera (i.e., *Bacteroides*, *Lactobacillus*, and *Peptoniphilus*) that were significantly associated with poor prognosis in multivariate analysis (hereafter referred to as “prognostic bacteria”) were all anaerobes. The abundance among these bacteria were shown in Fig. 4A. A low (*r* = 0.28) but significant positive correlation was found between the abundance of *Bacteroides* and *Lactobacillus* (*P* = 0.048). While there was no significant correlation between *Peptoniphilus* and *Bacteroides*, as well as *Peptoniphilus* and *Lactobacillus*, there was a positive trend in the abundance of both. Thus, we hypothesized that a hypoxic environment conducive to the growth of these prognostic bacteria may influence the progression of PDAC. To test this hypothesis, we compared clinical and tumor characteristics between 24 cases positive for at least one of the three prognostic bacteria and 28 cases negative for all of them (Table 2). There were no significant differences in clinical characteristics including the tumor size and pathological stage between the two groups. Features in tumor genomes such as alterations in *KRAS, TP53, CDKN2A/p16,* and *SMAD4* genes were also comparable between the two groups. Quantification of the area of tumor stromal collagen and αSMA^+^ fibroblasts showed no significant difference in intra-tumor fibrosis as well. In marked contrast, the degree of tumor-infiltrating T cells positive for CD4, CD8, and CD45RO were significantly lower in the tumors with prognostic bacteria than their counterparts (*P* = 0.005, *P* = 0.03, and *P* = 0.005, respectively). We also evaluated tumor-associated macrophage using CD68 and CD206 as markers of M1-like and M2-like macrophages, respectively, and found that the abundance of intra-tumor immunosuppressive M2 macrophages tended to be higher in the group with prognostic bacteria than their counterpart although the difference was not significant.

**Table 2:** Association between the presence of bacteria associated with poor prognosis and tumor characteristics in PDAC

	All cases	Presence of prognostic bacteria	Absence of prognostic bacteria	*P* value
	*N* = 52	*N* = 24	*N* = 28	
Tumor size (mm), *n* (%)				0.55
< 28	24 (46.2)	10 (41.7)	14 (50.0)	
≥ 28	28 (53.8)	14 (58.3)	14 (50.0)	
Pathological stage^a^, *n* (%)				0.09
< IIB	17 (32.7)	5 (20.8)	12 (42.9)	
≥ IIB	35 (67.3)	19 (79.2)	16 (57.1)	
CD4 (count/mm^2^), *n* (%)				0.005
Low (< 55)	26 (50.0)	17 (70.8)	9 (32.1)	
High (≥ 55)	26 (50.0)	7 (29.2)	19 (67.9)	
CD8 (count/mm^2^), *n* (%)				0.03
Low (< 108)	26 (50.0)	16 (66.7)	10 (35.7)	
High (≥ 108)	26 (50.0)	8 (33.3)	18 (64.3)	
FOXP3 (count/mm^2^), *n* (%)				0.37
Low (< 33)	26 (50.0)	15 (62.5)	14 (50.0)	
High (≥ 33)	26 (50.0)	9 (37.5)	14 (50.0)	
CD45RO (count/mm^2^), *n* (%)				0.005
Low (< 100)	26 (50.0)	17 (20.8)	9 (32.1)	
High (≥ 100)	26 (50.0)	7 (79.2)	19 (67.9)	
CD68 (count/mm^2^), *n* (%)				0.57
Low (< 89)	26 (50.0)	13 (54.2)	13 (46.4)	
High (≥ 89)	26 (50.0)	11 (45.8)	15 (53.6)	
CD206 (count/mm^2^), *n* (%)				0.09
Low (< 75)	26 (50.0)	9 (37.5)	17 (60.7)	
High (≥ 75)	26 (50.0)	15 (62.5)	11 (39.3)	
Tumor stromal collagen				0.10
Low	26 (50.0)	14 (58.3)	10 (35.7)	
High	26 (50.0)	10 (41.7)	18 (64.3)	
αSMA positive fibroblast				0.41
Low	26 (50.0)	13 (54.2)	12 (42.9)	
High	26 (50.0)	11 (45.8)	16 (57.1)	
*KRAS mutation*, *n* (%)				0.61
Absent	4 (7.7)	1 (4.2)	3 (10.7)	
Present	48 (92.3)	23 (95.8)	25 (89.3)	
*TP53 alteration*, *n* (%)				0.74
Absent	14 (26.9)	7 (29.2)	7 (25.0)	
Present	38 (73.1)	17 (70.8)	21 (75.0)	
*CDKN2A/p16 alteration*, *n* (%)				0.48
Absent	20 (38.5)	8 (33.3)	12 (42.9)	
Present	32 (61.5)	16 (66.7)	16 (57.1)	
*SMAD4 alteration*, *n* (%)				0.97
Absent	28 (53.8)	13 (54.2)	15 (53.6)	
Present	24 (46.2)	11 (45.8)	13 (46.4)	

*α-SMA* alpha-smooth muscle actine, *PDAC* pancreatic ductal carcinoma

^a^Pathological stage was classified according to the UICC 8th edition

The comparison of overall survival between the groups with and without prognostic bacteria confirmed that the survival of the group with prognostic bacteria was significantly poorer than that without prognostic bacteria (*P* < 0.001, Fig. 4B). Multivariate COX hazard analysis showed that the presence of prognostic bacteria was an independent prognostic factor (*P* < 0.001, HR = 3.48, 95%CI 1.65–7.33, adjusted for clinical characteristics as follow: prognostic bacteria, pathological stage and residual tumor status). These findings suggested that the presence of the prognostic bacteria was associated with suppressed anti-tumor immunity leading to poor prognosis.

**Fig. 5:**
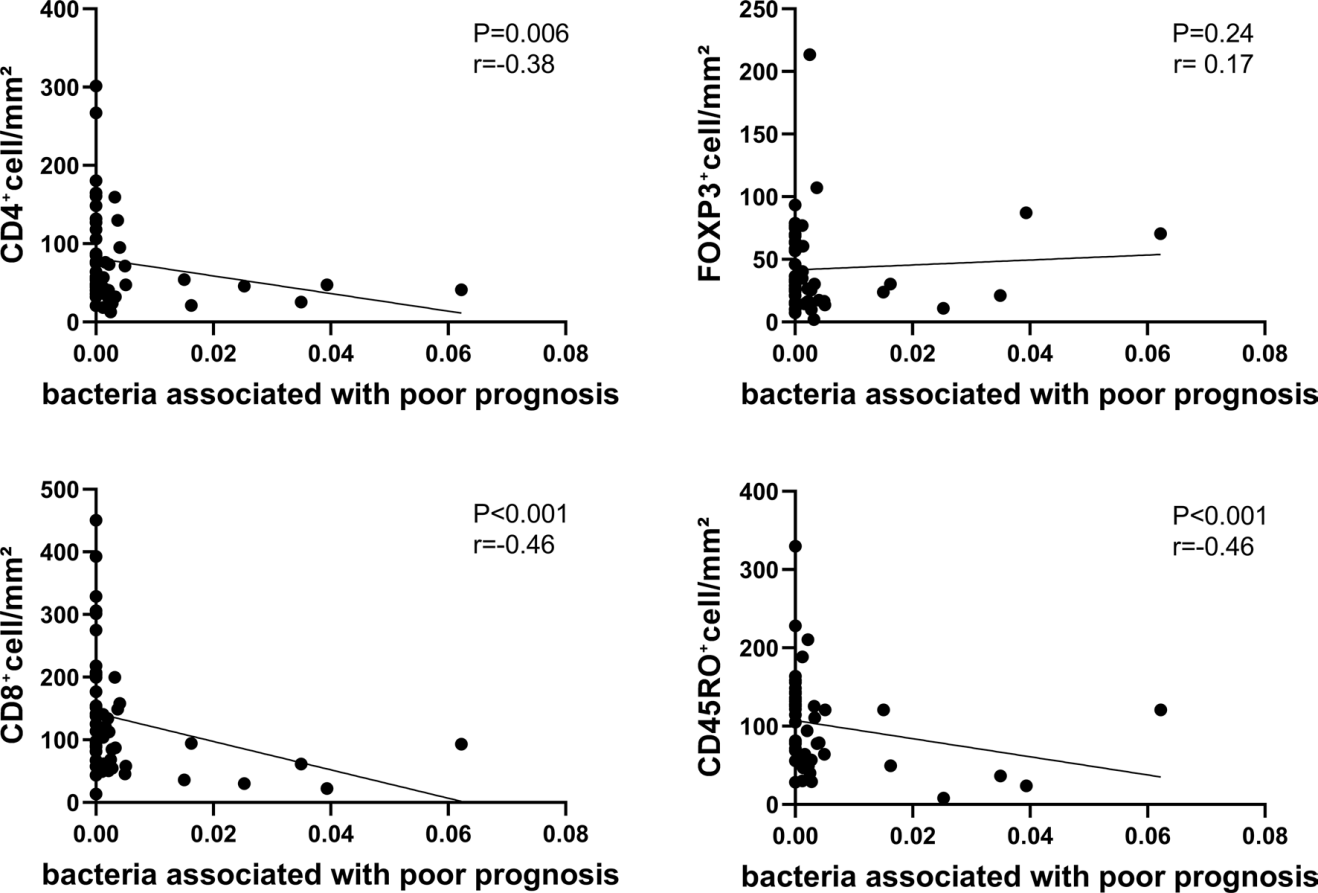
Correlation of the number of TILs and the presence of prognostic bacteria. The abundance of any of the genera *Bacteroides*, *Lactobacillus*, or *Peptoniphilus* was analyzed by Spearman correlation. *TILs* tumor-infiltrating lymphocytes

An in-depth investigation of the correlation between the presence of the prognostic bacteria and TILs was performed. The significant negative correlations were detected between the abundance of the prognostic bacteria and the number of CD4^+^, CD8^+^, CD45RO^+^ T cells in PDAC (*P* = 0.006, *P* < 0.001, *P* < 0.001, respectively, Fig. 5). Such significant negative correlations between the prognostic bacteria and tumor-suppressive TILs were also observed when each genus was examined (Supplementary Fig. 5); CD45 RO^+^ T cells in *Bacteroides* (*P* < 0.001), CD8^+^ and CD45RO^+^ T cells in *Lactobacillus* (*P* = 0.009, *P* = 0.007, respectively), and CD4^+^ and CD8^+^ cells in *Peptoniphilus* (*P* = 0.004, *P* = 0.017, respectively). On the contrary, there was no significant correlation between the amount of immune-suppressive FOXP3^+^ T cells infiltrating in tumors and the abundance of the prognostic bacteria (Fig. 5). These findings support the fact that TME with the three prognostic bacteria is associated with the suppression of anti-tumor immunity and poor prognosis.

## Discussion

In this study, we observed a correlation between the presence of three anaerobic bacteria, *Bacteroides*, *Lactobacillus*, and *Peptoniphilus*, within PDAC with a suppressed anti-tumor immunity, and poor prognosis. In contrast, there was no significant correlation between these bacteria and intratumoral fibrosis or alterations in major driver genes in tumor genomes.

In recent years, the presence of microbiome in PDAC has been demonstrated by several studies [18]. We showed that bacteria within PDAC exhibited a distinct profile from that within non-tumoral pancreatic tissues and were predominantly composed of the phyla *Proteobacteria* and *Firmicutes*, which was consistent with previous reports [10, 19]. Although it has been controversial whether the bacterial profile within PDAC is different from that in non-tumoral pancreatic tissues [8, 9, 20], comparative analysis using samples that exhibited the evidence of bacterial presence in the present study demonstrated the colonization of distinct bacterial species within PDAC that were different from those in the surrounding tissue. The unique TME of PDAC such as extensive desmoplasia leading to hypoxia may allow the colonization of a characteristic microbiome that is distinct from the surrounding tissue environment [21]. A microbiota analysis with a larger cohort would be necessary to draw definitive conclusions.

Intratumoral infiltration of immune cells plays a crucial role in regulating tumor progression [22–24]. We previously demonstrated a positive correlation between increased infiltration of CD4^+^, CD8^+^, and CD45RO^+^ T cells within the PDAC and favorable prognosis of PDAC patients [17]. Importantly, several studies have reported that the gut and intra-tumor microbiome are involved in the regulation of intratumoral immune ecosystem in various malignancies including PDAC [8, 11, 25–28], although, the precise role of the microbiome in PDAC remains largely unclear. In the present study, we demonstrated that the increased abundance of three anaerobic bacteria including *Bacteroides*, *Lactobacillus*, and *Peptoniphilus* within PDAC was significantly correlated with the suppressed intratumoral infiltration of effector T cells as well as a poor prognosis of PDAC. PDAC is well-known to create a hypoxic microenvironment due to its limited cellularity and compressed, desmoplastic stroma [29, 30]. Hypoxic conditions in PDAC reportedly enhanced the intracellular survival of an anaerobic bacteria, *Porphyromonas gingivalis* [31]. Notably, a recent study showed that the hypoxic environment was enhanced by intestinal bacteria vice versa and modulated tissue-resident lymphocytes in mice [32]. Moreover, some anaerobic bacteria such as *Fusobacterium nucleatum* are associated with immune suppression [33]. Thus, tumor stroma as well as intratumoral colonization of bacteria in itself, may establish the hypoxic microenvironment that allows the growth of anaerobic bacteria in a subset of PDAC, which might lead to poor prognosis via immune suppression. It also remains unclear whether the intra-tumor bacteria themselves are directly involved in suppressing the intra-tumor immune system. Tryptophan metabolites derived from Lactobacillus have recently been reported to induce immunosuppression by affecting intra-tumor macrophages [34]. In line with this, we found that intra-tumor immunosuppressive M2 macrophages tended to be higher in the prognostic bacteria group. Moreover, some studies have also reported direct involvement of certain bacteria in tumor immunosuppression. *F. nucleatum* within PDAC was demonstrated to promote pancreatic cancer progression through autocrine and paracrine mechanisms of the CXCL1–CXCR2 axis [35]. Intratumoral *P. gingivalis* was also reported to promote PDAC progression via elevating the secretion of neutrophilic chemokines and neutrophil elastase [36]. Further studies are warranted to investigate whether certain specific anaerobic bacteria are involved in inducing immune suppression through some mechanism or whether anaerobic bacteria just tend to colonize in PDAC with poor prognosis.

One of the major challenges in microbiome analysis using human pancreatic tissues is the inevitable presence of contamination, as it is difficult to aseptically extract bacteria from human pancreatic samples. By evaluating the effect of environmental contamination with data derived from non-tissue FFPE pieces and by analyzing only bacteria for which possibilities of contamination were statistically excluded [15], we addressed this issue and obtained unique bacterial profiles in tumoral and non-tumoral pancreatic tissues. Additionally, some clinical events such as preoperative endoscopic procedures might have affected the intra-PDAC microbiome in the present study. Indeed, endoscopic ultrasound-guided fine needle biopsy or endoscopic retrograde cholangiopancreatography was performed for diagnosis and drainage purpose in all cases analyzed. If endoscopic procedures could enhance the colonization of bacteria in PDAC tissues and intratumoral presence of anaerobic bacteria might lead to the suppression of anti-PDAC immunity, prophylaxis against such anaerobic bacteria during endoscopic procedures could be important to improve the treatment efficiency against PDAC.

In conclusion, our findings demonstrate that the presence of anaerobic genera such as *Bacteroides*, *Lactobacillus*, and *Peptoniphilus* within PDAC potentially have prognostic relevance. These genera might be implicated in immune-mediated prognostic deterioration of tumors. This study emphasizes the significance of anaerobic bacteria colonization in PDAC in the clinical management of PDAC, although further comprehensive investigations are necessary to fully understand its implications.

### Supplementary Information

Below is the link to the electronic supplementary material.

Supplementary file1 Supplementary Fig. 1 Representative images of immunohistochemistry and Elastica van Gieson staining showing high or low levels of CD4^+^, CD8^+^, FOXP3^+^, CD45RO^+^, CD68, CD206 and tumor stromal collagen. Scale bars, 50 μm. *TILs* tumor-infiltrating lymphocytes (TIF 89838 KB)

Supplementary file2 Supplementary Fig. 2 Microbiome composition in PDAC, non-tumoral tissues and non-tissue FFPE pieces. **A** Taxonomic profiles of predominant bacterial genera by mean relative abundance (%) in PDAC tissues, non-tumoral tissues, and non-tissue FFPE pieces before using Decontam. **B** Taxonomic profiles of predominant bacterial phylum by mean relative abundance (%) in PDAC and non-tumoral tissues after filtering with Decontam (TIF 14528 KB)

Supplementary file3 Supplementary Fig. 3 Diversity analysis of the group of administration of antibiotics and not administration of antibiotics. **A** Comparative analysis of the alpha diversity of the microbiome communities between the group of administration of antibiotics and no administration of antibiotics using Shannon index. **B** Principal coordinate analysis (PCoA) using weighted-UniFrac distance of beta diversity between the group of administration of antibiotics and no administration of antibiotics. *PDAC* pancreatic ductal adenocarcinoma (TIF 4608 KB)

Supplementary file4 Supplementary Fig. 4 Diversity analysis of long-term survival group and short-term survival group. **A** Comparative analysis of the alpha diversity of the microbiome communities between long-term and short-term survival groups using Shannon index. **B** Principal coordinate analysis (PCoA) using weighted-UniFrac distance of beta diversity between long-term and short-term survival groups. *PDAC* pancreatic ductal adenocarcinoma (TIF 4515 KB)

Supplementary file5 Supplementary Fig. 5 Correlation between TME findings and the abundance of *Bacteroides*, *Lactobacillus*, and *Peptoniphilus* in tumors. *P* values and correlation coefficient (*r*) values were analyzed by Spearman correlation. *TME* tumor microenvironment, *TILs* tumor-infiltrating lymphocytes, *α-SMA* alpha-smooth muscle actine (TIF 6828 KB)

Supplementary file6 (DOCX 32 KB)
