# Supplementary material for: Impact of intratumoral microbiome on tumor immunity and prognosis in human pancreatic ductal adenocarcinoma
Source: J Gastroenterol. 2024 Jan 20;59(3):250–62. doi: 10.1007/s00535-023-02069-5 (PMC10904450; doi:10.1007/s00535-023-02069-5)
Supplement: Supplementary file 6 — Supplementary file6 (DOCX 32 KB) [file 535_2023_2069_MOESM6_ESM.docx]

**Supplementary table 1. Comparison of patients’ characteristics of short-term and long-term survival groups in PDAC**

|  | **All cases** | **Short term survival group** | **Long term survival group** | ***P* value** |
| --- | --- | --- | --- | --- |
|  | **N=52** | **N=25** | **N=27** |  |
| Age (years), median (range) | 69 (50-83) | 69(50-82) | 69 (53-83) | 0.23 |
| Gender, n (%) |  |  |  | 0.25 |
| Male | 29 (55.7) | 16 (64.0) | 13 (48.1) |  |
| Female | 23 (44.3) | 9 (36.0) | 14 (51.9) |  |
| BMI (kg/m^2^), median (range) | 20.9 (17.1-33.2) | 21.0 (17.1-33.2) | 21.1 (17.2-26.8) | 0.80 |
| CEA (ng/ml), mean (range) | 3.1 (0.1-68.8) | 3.3 (0.4-68.8) | 3.1 (1.2-33.6) | 0.34 |
| CA19-9 (U/ml), mean (range) | 149 (1-11973) | 149 (1-11973) | 144 (1-1346) | 0.13 |
| White blood cell count (/µL), median (range) | 5400 (3600-8600) | 5400 (3800-8800) | 5300 (3600-7500) | 0.19 |
| Neutrophil count (/µL), median (range) | 3199 (2113-6879) | 3199 (2246-5570) | 3116 (2113-6879) | 0.10 |
| Total leukocytes count (/µL), median (range) | 1527 (749-2580) | 1528 (806-2369) | 1513 (750-2580) | 0.58 |
| Alcohol consumption, n (%) |  |  |  | 0.61 |
| Absent | 48 (92.3) | 24 (96.0) | 24 (88.9) |  |
| Present | 4 (7.7) | 1 (4.0) | 3 (11.1) |  |
| Current smoking, n (%) |  |  |  | 0.89 |
| Absent | 42 (80.8) | 20 (80.0) | 22 (81.5) |  |
| Present | 10 (19.2) | 5 (20.0) | 5 (18.5) |  |
| Diabetes mellitus, n (%) |  |  |  | 0.27 |
| Absent | 27 (51.9) | 11 (44.0) | 16 (59.3) |  |
| Present | 25 (48.1) | 14 (56.0) | 11 (40.7) |  |
| Tumor size (mm), median (range) | 29 (12-45) | 29 (12-44) | 29 (13-45) | 0.088 |
| Tumor location, n (%) |  |  |  | 0.14 |
| Head | 35 (67.4) | 20 (80.0) | 15 (55.6) |  |
| Body | 15 (28.8) | 4 (16.0) | 11 (40.7) |  |
| Tail | 2 (3.8) | 1 (4.0) | 1 (3.7) |  |
| Pathological stage^#^, n (%) |  |  |  | 0.01 |
| IA | 7 (13.5) | 0 | 7 (25.9) |  |
| IB | 5 (9.6) | 1 (4.0) | 4 (14.8) |  |
| IIA | 5 (9.6) | 3 (12.0) | 2 (7.4) |  |
| IIB | 28 (53.8) | 15 (60.0) | 13 (48.1) |  |
| III | 7 (13.5) | 6 (24.0) | 1 (3.8) |  |
| Histological grade, n (%) |  |  |  | 0.46 |
| Well | 16 (30.8) | 6 (24.0) | 10 (37.0) |  |
| Moderate | 33 (63.5) | 18 (72.0) | 15 (55.6) |  |
| Poorly | 3 (5.7) | 1 (4.0) | 2 (7.4) |  |
| Residual tumor status, n (%) |  |  |  | 0.01 |
| R0 | 38 (73.1) | 14 (56.0) | 24 (88.9) |  |
| R1 | 14 (26.9) | 11 (44.0) | 3 (11.1) |  |
| Neoadjuvant chemotherapy, n (%) | 5 (9.6) | 3 (12.0) | 2 (7.4) | 0.66 |
| Adjuvant chemotherapy, n (%) | 37 (71.2) | 16 (64.0) | 21 (77.8) | 0.27 |

^#^Pathological stage was classified according to the UICC 8th edition.BMI, body mass index; CEA, carcinoembryonic antigen; CA19-9, carbohydrate antigen 19-9; PDAC, pancreatic ductal carcinoma

**Supplementary table 2. Presence of intra-tumor bacteria and patient mortality in PDAC**

| **Intra-tumor bacteria**  **(genera)** |  | **No. of cases** | **No. of event** | **Univariate HR (95%CI)** | ***P* value** | **Multivariate HR^#^ (95%CI)** | ***P* value** |
| --- | --- | --- | --- | --- | --- | --- | --- |
| Fusicatenibacter | Absent | 40 | 28 | 1 (reference) | 0.03 | 1 (reference) | 0.97 |
|  | Present | 12 | 10 | 2.22 (1.07-4.63) |  | 0.91 (0.18-5.35) |  |
| Unknown Lactobacillales | Absent | 48 | 34 | 1 (reference) | 0.03 | 1 (reference) | 0.15 |
|  | Present | 4 | 4 | 3.36 (1.13-10.0) |  | 3.12 (0.65-15.0) |  |
| Peptoniphilus | Absent | 43 | 29 | 1 (reference) | 0.001 | 1 (reference) | 0.01 |
|  | Present | 9 | 9 | 2.73 (1.25-5.91) |  | 4.62 (1.86-11.5) |  |
| Unknown Neisseriaceae | Absent | 44 | 30 | 1 (reference) | 0.02 | 1 (reference) | 0.62 |
|  | Present | 8 | 8 | 2.58 (1.14-5.87) |  | 1.33 (0.44-4.04) |  |
| Roseburia | Absent | 35 | 23 | 1 (reference) | 0.008 | 1 (reference) | 0.71 |
|  | Present | 17 | 15 | 2.46 (1.27-4.76) |  | 1.27 (0.4-4.6) |  |
| Oscillospiraceae;UCG-005 | Absent | 43 | 30 | 1 (reference) | 0.001 | 1 (reference) | 0.17 |
|  | Present | 9 | 8 | 3.75 (1.66-8.46) |  | 3.6 (0.58-22.8) |  |
| Lactobacillus | Absent | 38 | 25 | 1 (reference) | <0.001 | 1 (reference) | 0.002 |
|  | Present | 14 | 13 | 3.31 (1.64-6.65) |  | 4.06 (1.71-9.70) |  |
| Muribaculaum | Absent | 48 | 34 | 1 (reference) | 0.01 | 1 (reference) | 0.40 |
|  | Present | 4 | 4 | 4.36 (1.41-13.5) |  | 0.48(0.08-2.71) |  |
| Unknown Lachnospiraceae | Absent | 39 | 27 | 1 (reference) | 0.03 | 1 (reference) | 0.41 |
|  | Present | 13 | 11 | 2.19 (1.07-4.46) |  | 0.55 (0.13-2.27) |  |
| Bacteroides | Absent | 43 | 29 | 1 (reference) | <0.001 | 1 (reference) | 0.01 |
|  | Present | 9 | 9 | 4.89 (2.19-10.9) |  | 5.95 (1.49-23.9) |  |
| *Staphylococcus* | Absent | 42 | 31 | 1 (reference) | 0.23 | - |  |
|  | Present | 10 | 7 | 1.67 (0.73-3.81) |  | - |  |

# The hazard ratio was adjusted for genera that were significantly different in the univariate analysis.

HR, hazard ratio; PDAC, pancreatic ductal carcinoma
